# Human Immunodeficiency Virus Type 1 Vif Up-Regulates the Expression of Tat *via* AKT Signaling Pathway: Role of Ubiquitin Specific Protease 17

**DOI:** 10.3389/fmicb.2022.828430

**Published:** 2022-03-21

**Authors:** Sneh Lata, Vikas Sood, Akhil C. Banerjea

**Affiliations:** ^1^Virology II, National Institute of Immunology, New Delhi, India; ^2^Department of Biochemistry, Jamia Hamdard University, New Delhi, India

**Keywords:** HIV-1, Tat, AKT, Vif, phosphorylation, DUB, Mdm2, USP17

## Abstract

Human immunodeficiency virus type 1 (HIV-1) has RNA genome and depends on host cellular machinery for most of its activities. Host cellular proteins modulate the expression and activity of viral proteins to combat the virus. HIV-1 proteins are known to regulate each other for the benefit of virus by exploiting these modulations. Here, we report that HIV-1 Vif increases the levels of Tat *via* AKT signaling pathway. We show that HIV-1 Vif activates AKT signaling pathway by inducing phosphorylation of AKT. Mdm2, downstream target of AKT signaling, increases the levels of Tat protein in ubiquitin-dependent manner by inducing Ubiquitin Specific Protease 17 (USP17), which is a deubiquitinase and stabilizes Tat protein. Thus, HIV-1 proteins exploit AKT signaling pathway to promote viral replication.

## Introduction

Human immunodeficiency virus type 1 (HIV-1) is a retrovirus having two copies of single-stranded RNA genome. It encodes for three structural proteins, namely, Gag, Pol, and Env, and six accessory proteins, namely, Tat, Rev, Vif, Vpr, Vpu, and Nef (Frankel and Young, 1998). These proteins interact with many cellular proteins and hijack cellular machinery for promoting viral replication by overcoming various host restriction factors (Emerman and Malim, 1998; Malim and Emerman, 2008). HIV-1 Nef down-regulates CD4 (cluster of differentiation 4) (Chaudhuri et al., 2007) and MHC-1 (major histocompatibility complex 1) (Blagoveshchenskaya et al., 2002) to evade innate immune recognition and to prevent superinfection. HIV-1 Vif down-regulates APOBEC3G (apolipoprotein B mRNA-editing enzyme-catalytic polypeptide-like 3G) by proteasomal degradation (Yu et al., 2003). Vpu down-regulates tetherin in β-TrCP (β-transducin repeat containing protein)–dependent manner (Douglas et al., 2009).

Human immunodeficiency virus type 1 is known to induce the activation of PI3/AKT signaling pathway, and this effect is mediated by Tat protein (Borgatti et al., 1997; Chugh et al., 2001, 2008; Deregibus et al., 2002). Mdm2 is a downstream target of AKT (Protein Kinase B; Cooray, 2004; Manning and Cantley, 2007). Previously, we have shown that HIV-1 Tat protein stabilizes Mdm2 by inducing its phosphorylation in AKT-dependent manner (Raja et al., 2017). MDM2 is also known to enhance the Tat-mediated LTR activity. MDM2 ubiquitinates Tat at lysine 71 position to potentiate its activity in a non-proteolytic way (Bres et al., 2003). Thus, there is a positive feedback loop between Tat, AKT, and Mdm2. However, MDM2 is an E3 ligase that has been shown to interact with HIV-1 Vif, leading to its ubiquitination followed by its proteasomal degradation (Izumi et al., 2009). Vif protein is required for efficient viral replication because it counteracts the host restriction factor APOBEC3G (Zhang et al., 2003; Goila-Gaur and Strebel, 2008). APOBEC3G induces hypermutations in viral DNA by cytidine deaminase activity leading to degradation of viral DNA. If hypermutated viral DNA gets integrated in the cellular genome, it cannot code for functional viral proteins (Zhang et al., 2003). Vif recruits E3 ubiquitin ligase complex and decreases the levels of APOBEC3G protein *via* proteasomal degradation pathway (Yu et al., 2003). Vif-deficient viruses are severely compromised and unable to multiply in host cells (Goila-Gaur and Strebel, 2008).

So, the effect of HIV-1 Vif expression on AKT signaling pathway was examined to investigate the mechanism how HIV-1 escapes the host antiviral response of Mdm2-mediated degradation of Vif protein. We found that Vif increased the phosphorylation of AKT on the one hand, whereas it induced ubiquitin-mediated proteasomal degradation of Mdm2 on the other hand. As Tat is previously known to activate AKT signaling pathway (Borgatti et al., 1997; Chugh et al., 2001, 2008; Deregibus et al., 2002), and HIV-1 proteins are also known to regulate each other by modulating the function of host proteins and support the viral replication; namely, Vif degrades Vpr protein and reduces Vpr-mediated cell cycle arrest (Wang et al., 2008); Rev induces the degradation of Tat in ubiquitin-independent manner *via* regulation of NQO1 [NAD(P)H: quinone oxidoreductase 1] (Lata et al., 2015), so we investigated the effect of Vif on the expression of Tat to find out how HIV-1 is benefited by activating AKT signaling pathway *via* its two proteins, Tat and Vif, despite the fact that Mdm2, which is activated by AKT, degrades Vif protein. We found that Vif increased the levels of Tat protein. Vif was also found to increase the LTR transcription mediated by Tat protein. Inhibition of AKT phosphorylation abrogated Vif-mediated increase in levels of Tat protein. Here, Mdm2 (target of AKT) was found to increase the levels of Tat *via* a deubiquitinase, Ubiquitin Specific Protease 17 (USP17). Thus, AKT signaling pathway was playing an important role in the regulation of HIV-1 Tat by Vif *via* Mdm2 mediated stabilization of USP17. This study can have significant implications toward better understanding of the several mechanisms of HIV-1–mediated exploitation of host machinery and viral pathogenesis.

## Materials and Methods

### Cell Culture and Transfection

Human embryonic kidney 293T (HEK-293T) and Tzm-Bl cells were maintained in Dulbecco modified eagle medium (Himedia Laboratories, India) supplemented with 10% fetal bovine serum (Gibco, Invitrogen, United States), 100 units of penicillin, 0.1 mg streptomycin, and 0.25 μg amphotericin B per ml at 37°C in the presence of 5% CO_2_ in a humidified incubator. THP-1, U937, and U1 cells were maintained in RPMI-1640 supplemented with 10% fetal bovine serum (Gibco, Invitrogen, United States), 100 units of penicillin, 0.1 mg streptomycin, and 0.25 μg amphotericin B (Himedia Laboratories, India) per milliliter at 37°C in the presence of 5% CO_2_ in a humidified incubator. Transfections were performed using Lipofectamine 2000 (Invitrogen, United States) and polyethylenimine, Linear (MW 25,000, Polysciences Inc., United States) reagents using the manufacturer’s protocol.

### Plasmid Constructs and Chemicals

Plasmid Myc Vif was made by cloning pNL4-3–derived gene *vif* in pCMV-Myc plasmid from Clontech, United States, as described earlier (Arora et al., 2014). pBlue3′LTR-luc was obtained from NIH AIDS Reference and Reagent Program of NIH, MD, United States. Glutathione S-transferase (GST) Tat was generated by cloning pNL4-3 derived *tat* gene in pGEX-4T1 vector from Addgene. HA Tat and Flag Tat were purchased from Addgene. HA Mdm2 was purchased from Sino Biologicals, United States. HA AKT, HA KD AKT (K179A), and HA Myr AKT were kind gifts from Hui Kuan Lin, MD Anderson Cancer Center, TX, United States. Renilla luciferase plasmid was a kind gift from Vivek Natrajan, IGIB, Delhi, India. His Ub plasmid was gifted by Dimitris Xirodimas, University of Dundee. Chemicals used were AKTi (Sigma, United States), PMA (Sigma, United States), IPTG (Sigma, United States), cycloheximide (Sigma, United States), and MG132 (Sigma, United States).

### Western Blot Analysis

Human embryonic kidney 293T cells were transfected with gene of interest for 24 h. The cells were harvested and lysed in RIPA lysis buffer (1% NP-40, 20 mM TrisCl, pH 7.5, 150 mM NaCl, 1 mM Na_2_EDTA, 1 mM EGTA, 1% sodium deoxycholate, 1 mM Na_3_VO_4_). Protein estimation was carried out using BCA Protein Assay Kit (Pierce, Thermo Scientific, United States). An equal amount of protein was loaded on sodium dodecyl sulfate–polyacrylamide gel electrophoresis (SDS-PAGE) and was transferred to nitrocellulose membrane. The membranes were blocked with 5% non-fat dry milk (Himedia Laboratories, India). The primary antibodies used were anti-AKT, anti-Mdm2, anti-AKT substrate, anti-GAPDH, anti–phospho-AKT (S473) (Cell Signaling Technology), anti-Myc, anti-HA (Clontech), anti-GST (Santa Cruz Biotechnology), and anti-Vif (NIH, MD, United States). The secondary antibodies used were anti-rabbit/mouse–horseradish peroxidase–conjugated (Jackson ImmunoResearch). Blots were developed using ECL (enhanced chemiluminescence) reagent.

### Cycloheximide Chase Assay

To study the degradation kinetics of proteins, cycloheximide chase assay was performed. HEK-293T cells were transfected with gene of interest for 24 h and treated with CHX (100 μg/mL; Sigma). Cell lysates were prepared at indicated time points and subjected to 10% SDS-PAGE followed by Western blot analysis as described above.

### *In vitro* Ubiquitination Assay

*In vivo* ubiquitination assay was performed to detect ubiquitylated proteins in transfected mammalian cells. HEK-293T cells were cotransfected with plasmid encoding desired gene and His-Ub (6 × histidine–ubiquitin) for 24 h. After 24 h of transfection, 20 μM MG132 (Sigma-Aldrich) was added, and the cells were further incubated for 8 h. The cells were lysed in buffer A (6 M guanidinium-HCl, 0.1 M Na_2_HPO_4_/NaH_2_PO_4_, 10 mM imidazole; pH 8.0), sonicated, and centrifuged. Ni-NTA beads were added to the supernatant, and the mixture was incubated at room temperature for 6 h while rotating. Subsequently, the beads were washed with buffer A and buffer TI (25 mM Tris, pH 6.8, 20 mM imidazole). The ubiquitinated proteins were eluted in buffer containing 200 mM imidazole, 5% SDS, 0.15 M Tris, pH 6.7, 30% glycerol, and 0.72 M β-mercaptoethanol. The eluates were resolved by SDS-PAGE followed by Western blot analysis.

### Glutathione S-Transferase Fusion Protein Expression and Purification

pGEX-4T1 vector containing the desired gene was transformed in BL21 strain of *Escherichia coli* for expression and subsequent purification. The bacterial culture was induced with 0.5 mM IPTG at 16°C for 16 h. The cells were lysed by adding lysozyme (1 mg/mL) at 4°C with gentle shaking. DTT was added to the bacterial lysate after lysozyme treatment (100 μL of 1 M DTT). This was followed by sonication and extraction of proteins with Triton X-100. The solution was centrifuged at 12,000 revolutions/min (rpm) for 15 min at 4°C. The supernatant was then used for binding to glutathione beads at 4°C for 3 h. The beads were centrifuged at 2,500 rpm for 2 min at 4°C. The beads were washed until the supernatant stopped giving color with Bradford reagent. A control with GST only, bound to glutathione beads, was expressed and purified in similar way.

### Glutathione S-Transferase Pull-Down Assay

GST alone and GST-tagged proteins were expressed and purified as described previously. HEK-293T cells were transfected with gene of interest for 36 h. The cells were lysed in RIPA buffer. Ten micrograms of GST-tagged protein was incubated with the cell lysate at 4°C for 4 h. After incubation, the supernatant was discarded, and the beads were washed five to six times with chilled 1 × phosphate-buffered saline (PBS). The beads were boiled in Laemmli buffer and subjected to SDS-PAGE followed by immunoblotting with anti-Myc and anti-GST antibodies.

### Coimmunoprecipitation

The protein–protein interaction was studied by coimmunoprecipitation. The genes of interest were cotransfected in HEK-293T cells for 24 h. For affinity tag-based immunoprecipitation, cell lysates were prepared in CelLytic M and cell lysis reagent (Sigma) and were incubated with anti-Myc agarose beads (Sigma) at 4°C overnight. The beads were washed with IP buffer (Sigma). The purified protein complex bound to anti-Myc agarose beads was resolved by SDS-PAGE and subjected to Western blot analysis. Pierce™ Direct IP kit (Thermo Scientific) was used to pull down a protein without tag. HEK-293T cells were transfected with desired plasmids. After 24 h of transfection, cells were harvested and washed with PBS. Cells were lysed in cell lysis buffer, and antibody-conjugated agarose resin was added. It was rotated overnight at 4°C. After incubation, resin was pelleted and washed with wash buffer. The immunoprecipitated proteins were eluted using elution buffer. The aqueous solution containing eluted proteins was boiled with SDS–PAGE loading buffer for 5 min and analyzed by Western blotting.

### Dual-Luciferase Reporter Assay

Luciferase reporter assay was performed using the dual-luciferase reporter assay kit (Promega, United States). HEK-293T cells were cotransfected with luciferase reporter plasmid and the plasmids encoding genes of interest. Renilla luciferase was used as control to normalize the transfection efficiency. Empty pcDNA3.1 vector was used to equalize the amount of DNA transfected in each well. After 24 h of transfection, cell were harvested and lysed in lysis buffer (Promega, United States). Luciferase activity was measured by luminometer (Tecan, Switzerland) using two substrates (Promega, United States): one for firefly luciferase and another for Renilla luciferase (mixed with Stop and Glo buffer). The readings of firefly luciferase activity were normalized with those of Renilla luciferase activity to get the true luciferase reporter activity.

### Statistical Analysis

All the experiments were repeated three to four times. Results obtained are presented as mean ± standard error of the mean (s.e.m). *p*-values were calculated by a two-tailed *t*-test. Only values with *p* < 0.05 were considered significant.

## Results

### Vif Increases Phosphorylation of AKT at Ser473

**FIGURE 1:**
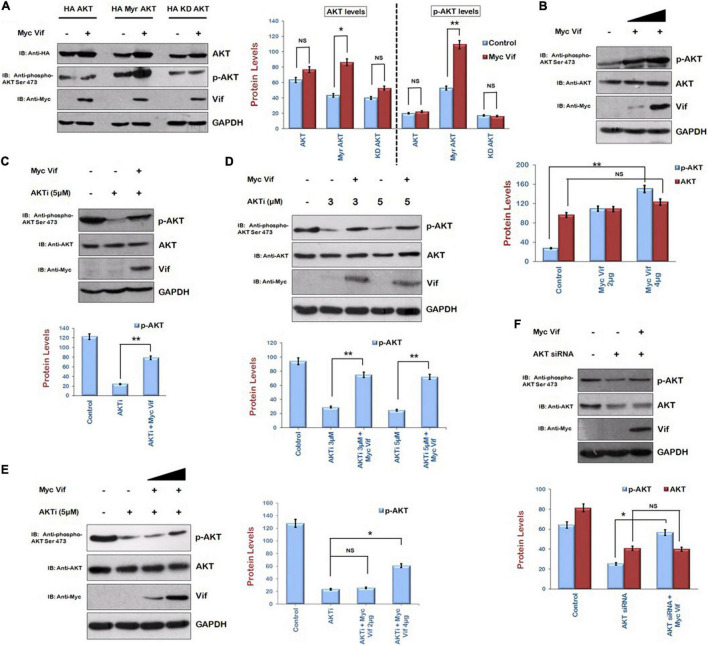
Human immunodeficiency virus type 1 Vif induces phosphorylation of AKT at Ser473. **(A)** HEK-293T cells were cotransfected with HA AKT, HA Myr-AKT, HA KD-AKT, and Myc Vif as shown for 24 h. Cell lysates were analyzed by Western blotting with anti-HA, anti–phospho-AKT Ser473, anti-Myc, and anti-GAPDH antibodies. **(B)** HEK-293T cells were transfected with increasing amounts of Myc Vif expression plasmid for 24 h. Cell lysates were analyzed by Western blotting with anti-AKT, anti-Myc, anti–phospho-AKT Ser473, and anti-GAPDH antibodies. **(C)** HEK-293T cells were transfected with Myc Vif expression plasmid and treated with AKTi (5 μM) as indicated for 24 h. Cell lysates were analyzed by Western blotting with anti-AKT, anti-Myc, anti–phospho-AKT Ser473, and anti-GAPDH antibodies. **(D)** HEK-293T cells were transfected with Myc Vif and treated with increasing dose of AKTi (3 and 5 μM) for 24 h. Cell lysates were analyzed by Western blotting with anti-AKT, anti-Myc, anti–phospho-AKT Ser473, and anti-GAPDH antibodies. **(E)** HEK-293T cells were treated with AKTi (5 μM) and transfected with increasing amount of Myc Vif (2 and 4 μg) for 24 h. Cell lysates were analyzed by Western blotting with anti-AKT, anti-Myc, anti–phospho-AKT Ser473, and anti-GAPDH antibodies. **(F)** HEK-293T cells were transfected with AKT siRNA and Myc Vif. Cell lysates were analyzed by Western blotting with anti-AKT, anti-Myc, anti–phospho-AKT Ser473, and anti-GAPDH antibodies. GAPDH was used as loading control. Densitometry analysis was performed using ImageJ and shown as bar graph with error bars. Data obtained are presented as mean ± s.e.m. of three independent experiments. *p*-value was calculated by two-tailed *t*-test [*p < 0.05, **p < 0.01, NS (not significant)].

Human embryonic kidney 293T cells were transfected with HA AKT (wild type), HA Myr AKT (Myristoylated, constitutively active), and HA KD AKT (kinase deficient) along with Myc Vif to investigate the effect of Vif on AKT signaling pathway. After 24 h of transfection, cells were lysed and analyzed by Western blotting. Vif increased the levels of Myr AKT, whereas there was no effect of Vif on wild-type HA AKT as well as KD AKT. Vif also increased the levels of phospho-AKT Ser473 in Myr AKT and Myc Vif cotransfection experiment but not in that of wild-type AKT as well as KD AKT (Figure 1A). We also checked the endogenous levels of phospho-AKT Ser473 in the presence of Vif. HEK-293T cells were transfected with increasing amounts of Myc Vif encoding plasmid. After 24 h of transfection, cell lysates were analyzed by Western blotting using anti–phospho-AKT Ser 473 antibody. It was observed that Vif increased the levels of phospho-AKT Ser473 in dose-dependent manner, whereas the expression of unmodified AKT was unaffected by Vif (Figure 1B).

This observation was also confirmed by using inhibitor of AKT phosphorylation, that is, AKTi. HEK-293T cells were transfected with Myc Vif with or without AKTi treatment. After 24 h, cells were subjected to Western blot analysis. AKTi reduced the levels of phospho-AKT Ser473 as expected. In presence of Vif, the phosphorylation of AKT at Ser473 was partially rescued (Figure 1C). These results indicate that HIV-1 Vif increases the phosphorylation of AKT at Ser473. The effect of Vif on AKT phosphorylation was also investigated using different doses of AKTi. HEK-293T cells were treated with different doses of AKTi (3 and 5 μM) and transfected with Myc Vif for 24 h. We found that Vif induced phosphorylation of AKT at both doses of AKTi (Figure 1D). The dose-dependent effect of Vif on AKT phosphorylation in the presence of fix dose of AKTi was also investigated. It was found that a lower amount (2 μg) of Myc Vif was not able to induce phosphorylation of AKT in the presence of AKTi, whereas a higher amount (4 μg) of Myc Vif could do so (Figure 1E).

These results were further validated using AKT-specific siRNA. AKT was knocked down using its specific siRNA, and the effect of Vif on the levels of AKT as well as p-AKT was investigated. We could achieve only 50% knockdown of AKT with AKT siRNA. The levels of phosphor-AKT Ser 473 were also down-regulated. It was observed that there was no effect of Vif on AKT levels in the presence of AKT siRNA, whereas the levels of p-AKT were up-regulated in the presence of Vif as compared with the lane with AKT siRNA alone (Figure 1F).

### Vif Increases Levels of Tat and Exhibits Direct Interaction

**FIGURE 2:**
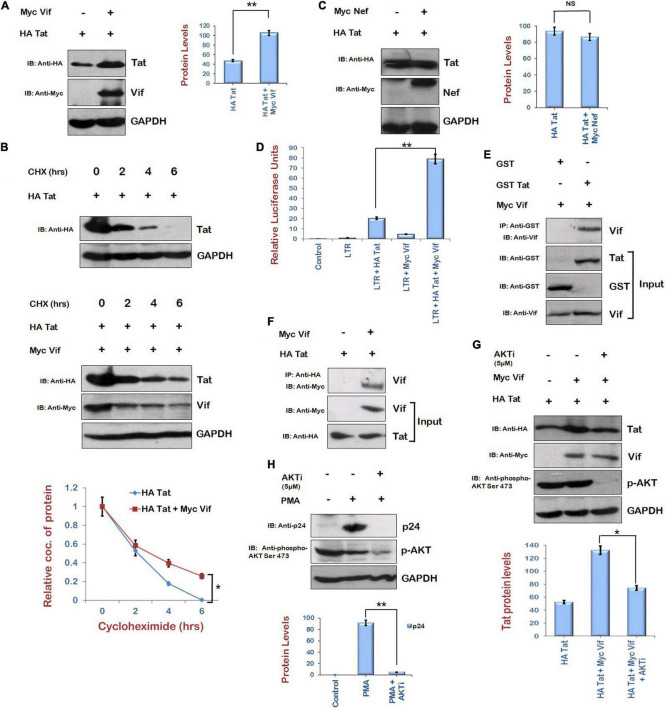
Human immunodeficiency virus type 1 Vif increases the expression of Tat *via* AKT signaling pathway. **(A)** HEK-293T cells were cotransfected with HA Tat and Myc Vif plasmids as indicated for 24 h. Cell lysates were analyzed by Western blotting using anti-HA, anti-Myc, and anti-GAPDH antibodies. **(B)** HEK-293 T cells were transfected with HA Tat either alone or along with Myc Vif for 24 h. Cells were treated with cycloheximide (100 μg/mL) for the indicated time periods. Cell lysates were subjected to Western blot analysis using anti-HA, anti-Myc, and anti-GAPDH antibodies. Densitometric analysis was done by ImageJ and shown as line graph with error bars. **(C)** HEK-293T cells were cotransfected with HA Tat and Myc Nef plasmids as indicated for 24 h. Cell lysates were analyzed by Western blotting using anti-HA, anti-Myc, and anti-GAPDH antibodies. **(D)** HEK-293T cells were cotransfected with HIV-1 LTR-luc reporter, Renilla luciferase, HA Tat, and Myc Vif expression plasmids as indicated for 24 h. Cells were lysed, and dual luciferase reporter assay was performed using luminometer. Relative luciferase activity is shown as bar graph. **(E)** GST Tat and GST alone bound with GST beads were incubated with *in vitro* synthesized Vif for 2 h at 4°C. Vif specific antiserum was used to probe the Vif protein in Western blot analysis. GST alone was used as negative control. **(F)** Tzm-Bl cells were transfected with HA Tat and Myc Vif plasmids for 24 h. Cell lysates were subjected to immunoprecipitation using anti-HA agarose beads at 4°C overnight. After incubation, beads were washed with IP buffer and boiled with PAGE loading buffer followed by SDS-PAGE and Western blotting using anti-Myc antibody. **(G)** HEK-293T cells were cotransfected with HA Tat and Myc Vif plasmids as indicated and treated with AKTi (5 μM) for 24 h. Cell lysates were analyzed by Western blotting using anti-HA, anti-Myc, anti–phospho-AKT Ser473, and anti-GAPDH antibodies. **(H)** HIV-1 replication in U1 cells was induced by PMA (100 μg/mL), and cells were treated with AKTi (5 μM) for 24 h. Cell lysates were analyzed by Western blotting using anti-p24, anti–phospho-AKT Ser473, and anti-GAPDH antibodies. GAPDH was used as loading control. Densitometry analysis was performed using ImageJ and shown as bar graph with error bars. Data obtained are presented as mean ± s.e.m. of three independent experiments. *p*-value was calculated by two-tailed *t*-test [*p < 0.05, **p < 0.01, NS (not significant)].

The effect of Vif on the levels of Tat was investigated by cotransfecting HEK-293T cells with HA Tat and Myc Vif for 24 h. Cell lysates were analyzed by Western blotting. Vif was found to stabilize the levels of Tat (Figure 2A). Cycloheximide chase of Tat either alone or with Vif also suggested that the stability of Tat is more in the presence of Vif (Figure 2B). As a control experiment, HA Tat was also coexpressed in HEK-293T cells along with Myc Nef, other protein of HIV-1. No effect of Nef was observed on the levels of Tat protein (Figure 2C). The effect of Vif on LTR transcription mediated by Tat was also investigated by cotransfecting HEK-293T cells with B LTR luciferase reporter plasmid (pBlue3′LTR-luc) and Tat either alone or in combination with Vif encoding plasmid for 24 h. As expected, LTR activation was observed in the presence of Tat. Vif alone also showed little activation of LTR. In the presence of both Vif and Tat proteins, LTR activation was observed to be much higher than that of Tat alone (Figure 2D), suggesting Vif-mediated increase in the levels of Tat protein.

The interaction between Tat and Vif was also investigated. *In vitro*–synthesized HIV-1 Vif protein and GST Tat fusion protein were used to study their interaction. For *in vitro* binding studies, GST Tat and GST alone (control) were allowed to bind to GST beads. Thereafter, *in vitro–*translated Vif protein was added to the complex and incubated for 2 h at 4°C. Vif protein was visualized on a nitrocellulose membrane by Western blot analysis using specific antiserum (obtained from NIH, MD, United States). GST Tat was able to pull down Vif, whereas GST alone failed to do so (Figure 2E). This suggests that Vif interacts with HIV-1 Tat protein. Further, we also investigated the intracellular interaction of Tat and Vif proteins *in vivo* in Tzm-Bl cells. For this, the cells were cotransfected with Myc Vif and HA Tat constructs. The cell lysates were prepared, and immunoprecipitation was carried out using anti-HA agarose (Sigma) followed by Western blot analysis using anti-Myc antiserum. It is clear from the Figure 2F that Tat interacted with Vif protein. These results suggest intracellular interaction of Tat and Vif proteins.

### Role of AKT Signaling Pathway in Human Immunodeficiency Virus Type 1 Vif–Mediated Stabilization of Tat

As Tat is known to play an important role in the activation of AKT signaling pathway (Borgatti et al., 1997; Chugh et al., 2001, 2008; Deregibus et al., 2002), and we are also reporting the up-regulation of phosphorylated AKT by Vif, the role of AKT signaling pathway in Vif-mediated stabilization of Tat levels was investigated using the chemical inhibitor of AKT phosphorylation (AKTi). HEK-293T cells were transfected with HA Tat either alone or with Myc Vif, and cells were treated with AKTi. Vif was observed to increase the levels of Tat protein as before, but inhibition of AKT phosphorylation by AKTi reduced the effect of Vif on the levels of Tat (Figure 2G). The effect of AKTi treatment on HIV-1 replication was also investigated in U1 cells (monocytes latently infected with HIV-1). HIV-1 replication was induced in U1 cells using PMA (100 μg/mL), and cells were treated with AKTi. The inhibition of AKT phosphorylation by AKTi was found to also reduce the HIV-1 replication (Figure 2H).

**FIGURE 3:**
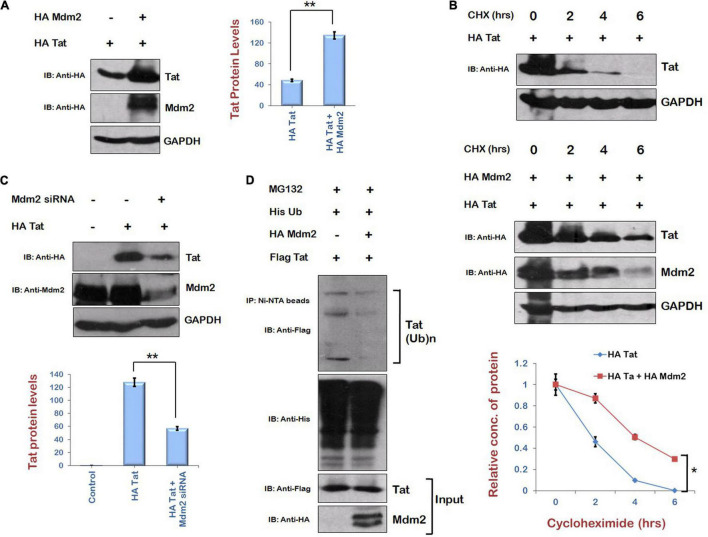
Mdm2 stabilizes the expression of HIV-1 Tat in ubiquitin-dependent manner. **(A)** HEK-293T cells were transfected with HA Tat either alone or along with HA Mdm2 for 24 h. Cell lysates were subjected to Western blot analysis using anti-HA and anti-GAPDH antibodies. **(B)** HEK-293 T cells were transfected with HA Tat either alone or along with HA Mdm2 for 24 h. Cells were treated with cycloheximide (100 μg/mL) for the indicated time periods. Cell lysates were subjected to Western blot analysis using anti-HA, anti-Myc, and anti-GAPDH antibodies. Densitometric analysis was done by ImageJ and shown as line graph with error bars. **(C)** HEK-293T cells were transfected with Mdm2 siRNA and HA Tat for 48 h. Cell lysates were subjected to Western blot analysis using anti-HA, anti-Mdm2, and anti-GAPDH antibodies. GAPDH was used as loading control. Densitometry analysis was performed using ImageJ and shown as bar graph with error bars. **(D)** HEK-293T cells were cotransfected with His Ub, Flag Tat, and HA Mdm2 for 24 h. Cells were treated with MG132 (20 μM) for 8 h. Cell lysates were subjected to immunoprecipitation with Ni-NTA beads followed by Western blotting with anti-Flag antibody. Data obtained are presented as mean ± s.e.m. of three independent experiments. *p*-value was calculated by two-tailed *t*-test (*p < 0.05, **p < 0.01).

As Mdm2 is the immediate downstream target of AKT signaling pathway and also known to mediate the K63 ubiquitination of Tat by direct interaction, resulting in the enhancement of LTR transcriptional activity of Tat and viral replication (Bres et al., 2003), the expression level of Tat in the presence of Mdm2 was examined. HEK-293T cells were cotransfected with HA Tat and HA Mdm2 for 24 h. The levels of Tat protein were found to be enhanced in the presence of Mdm2 (Figure 3A). Cycloheximide chase of Tat in the presence or absence of Mdm2 also showed that the stability of Tat was increased in the presence of Mdm2 (Figure 3B). The knockdown of Mdm2 using Mdm2-specific siRNA resulted in the down-regulation of Tat levels (Figure 3C). To investigate the mechanism of Mdm2-mediated up-regulation of HIV-1 Tat levels, the ubiquitination assay was performed. HEK-293T cells were transfected with Flag Tat, HA Mdm2, and His Ub plasmids for 24 h. Cells were treated with MG132 (proteasomal inhibitor) for 8 h followed by immunoprecipitation using Ni-NTA resin. Western blot analysis was done to detect the ubiquitinated species of Tat using anti-Flag antibody. The ubiquitination of Tat was found to be reduced in the presence of Mdm2 (Figure 3D). This result was in contradiction to the previous report that Mdm2 mediates K63 ubiquitination of Tat. K63 ubiquitination of a protein is known to increase the functional activity of protein rather than inducing its degradation in most of the cases (Bres et al., 2003; Wang et al., 2012). We have shown the total ubiquitination levels of Tat in the presence of Mdm2, which include ubiquitination linked to other lysine residues also (which leads to the degradation of protein). The total ubiquitination level of a protein is the net outcome of ubiquitination linked to different lysine residues. So, from the results in Figure 3D, we concluded that total ubiquitination of Tat is reduced in the presence of Mdm2 despite the fact that Mdm2 induces K63 ubiquitination of Tat. This might be the reason that the difference between the ubiquitination levels of Tat in the presence or absence of Mdm2 is less than expected. These results indicate that Mdm2 is the candidate protein of AKT signaling pathway, which increases the levels of Tat in ubiquitin-dependent manner.

### Regulation of Human Immunodeficiency Virus Type 1 Tat by Ubiquitin Specific Protease 17 in Ubiquitin-Dependent Manner

**FIGURE 4:**
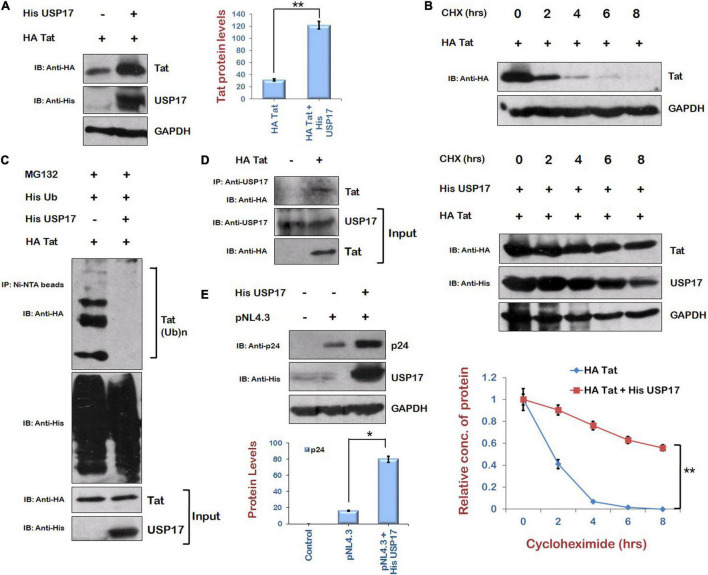
Ubiquitin Specific Protease 17 stabilizes the expression of Tat by deubiquitination. **(A)** HEK-293T cells were cotransfected with HA Tat and His USP17 plasmids for 24 h. Cell lysates were analyzed by Western blotting using anti-HA, anti-His, and anti-GAPDH antibodies. **(B)** HEK-293T cells were transfected with HA Tat either alone or along with His USP17 for 24 h. Cells were treated with cycloheximide (100 μg/mL) for the indicated time periods. Cell lysates were subjected to Western blot analysis using anti-HA, anti-His, and anti-GAPDH antibodies. Densitometric analysis was done by ImageJ and shown as line graph with error bars. **(C)** HEK-293T cells were cotransfected with His Ub, HA Tat, and His USP17 for 24 h. Cells were treated with MG132 (20 μM) for 8 h. Cell lysates were subjected to immunoprecipitation with Ni-NTA beads followed by Western blotting with anti-HA antibody. **(D)** HEK-293T cells were transfected with HA Tat encoding plasmid. After 24 h of transfection, cell lysates were subjected to immunoprecipitation using anti–USP17 antibody–bound agarose beads followed by Western blotting using anti-HA antibody. **(E)** HEK-293T cells were cotransfected with HA Tat and pNL4.3 for 24 h. Cell lysates were subjected to Western blotting with anti-HA, anti-p24, and anti-GAPDH antibodies. Densitometry analysis was performed using ImageJ and shown as bar graph with error bars. Data obtained are presented as mean ± s.e.m. of three independent experiments. *p*-value was calculated by two-tailed *t*-test (*p < 0.05, **p < 0.01).

As Mdm2 is stabilizing Tat levels in ubiquitin-dependent manner, and our laboratory has previously reported the stabilization of HIV-1 Tat protein in ubiquitin-dependent manner by USP7, which is a deubiquitinase (Ali et al., 2017), we explored the possibility of the involvement of a deubiquitinase in stabilization of Tat. We found a deubiquitinase, USP17, which is reported to promote cellular antiviral response by regulating virus-induced type 1 interferon signaling *via* deubiquitination of RIG-I and MDA5 (Chen et al., 2010). Thus, the effect of USP17 on HIV-1 Tat levels was investigated to find out how HIV-1 exploits USP17 for its own benefit to survive against host antiviral response. USP17 increased the levels of Tat when coexpressed in HEK-293T cells (Figure 4A). Cycloheximide chase of Tat in the presence of USP17 also indicated that stability of Tat is increased in the presence of USP17 (Figure 4B). Ubiquitination of Tat was also found to be decreased by USP17 (Figure 4C). These results indicate that USP17 stabilizes HIV-1 Tat by inducing its deubiquitination.

To check the interaction between Tat and USP17, HEK-293T cells were transfected with HA Tat, and coimmunprecipitation was performed using anti–USP17 antibody–bound agarose beads. This suggests that USP17 interacts with HIV-1 Tat protein (Figure 4D). To examine the functional consequences of USP17-mediated stabilization of Tat, the effect of USP17 on HIV-1 replication was also investigated by cotransfecting pNL4.3 and His USP17 in HEK-293T cells. USP17 was found to increase the viral replication as indicated by p24 expression (Figure 4E).

### Mdm2 Increases the Expression of Ubiquitin Specific Protease 17 *via* Direct Interaction

**FIGURE 5:**
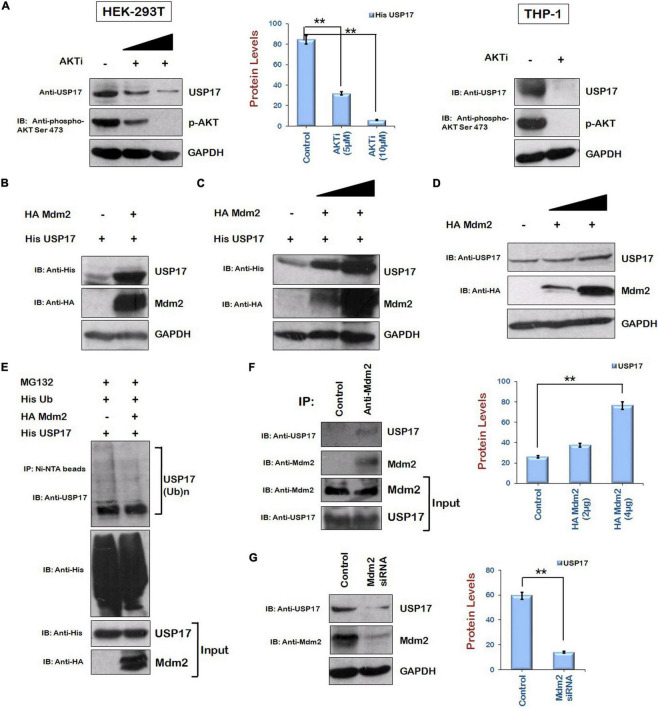
Mdm2 stabilizes the expression of USP17 in ubiquitin-dependent manner. **(A)** HEK-293T and Thp-1 cells were treated with AKTi as indicated for 24 h. Cell lysates were subjected to Western blotting using anti-USP17, anti–phospho-AKT Ser473, and anti-GAPDH antibodies. **(B)** HEK-293T cells were cotransfected with His USP17 and HA Mdm2 plasmids for 24 h. Cell lysates were analyzed by Western blotting using anti-His, anti-HA, and anti-GAPDH antibodies. **(C)** HEK-293T cells were transfected with His USP17 and increasing amounts of HA Mdm2 encoding plasmid. After 24 h of transfection, cell lysates were subjected to Western blotting using anti-His, anti-HA, and anti-GAPDH antibodies **(D)** HEK-293T cells were transfected with increasing amounts of HA Mdm2 plasmid for 24 h. Cell lysates were analyzed by Western blotting using anti-HA, anti-USP17, and anti-GAPDH antibodies. **(E)** HEK-293T cells were cotransfected with His Ub, His USP17, and HA Mdm2 for 24 h. Cells were treated with MG132 (20 μM) for 8 h. Cell lysates were subjected to immunoprecipitation with Ni-NTA beads followed by Western blotting with anti-USP17 antibody. **(F)** HEK-293T cell lysates were subjected to immunoprecipitation using anti–Mdm2 antibody–bound agarose beads followed by Western blotting using anti-USP17 antibody. **(G)** HEK-293 T cells were transfected with siRNA specific to Mdm2. After 48 h of transfection, cell lysates were subjected to Western blot analysis using anti-USP17, anti-Mdm2, and anti-GAPDH antibodies. Densitometry analysis was performed using ImageJ and shown as bar graph with error bars. GAPDH was used as loading control. Data obtained are presented as mean ± s.e.m. of three independent experiments. *p*-value was calculated by two-tailed *t*-test (**p < 0.01).

As HIV-1 Vif is increasing the levels of Tat protein *via* AKT signaling pathway in ubiquitin-dependent manner and USP17 stabilizes Tat by deubiquitination, the levels of USP17 in the presence of AKTi were examined to find out the role of AKT signaling pathway in regulating USP17 levels. Inhibition of AKT phosphorylation was found to reduce the levels of USP17 in HEK-293T cells. This result was further validated in THP-1 cells. AKTi treatment was found to reduce also the levels of USP17 in THP-1 cells (Figure 5A).

As we are showing that Mdm2 is the candidate protein of AKT signaling pathway, which is increasing Tat levels, the effect of Mdm2 on the expression of USP17 was also investigated by coexpressing them in HEK-293T cells. The levels of USP17 were found to be increased in the presence of Mdm2 (Figure 5B). The dose-dependent effect of Mdm2 on the levels of USP17 was also observed (Figure 5C). Mdm2 was also stabilizing USP17 at the endogenous level (Figure 5D). The ubiquitination of USP17 in the presence of Mdm2 was also investigated. HEK-293T cells were cotransfected with His USP17, HA Mdm2, and His Ub plasmids. Cells were also treated with MG132 to accumulate ubiquitinated species. Cells were lysed, and cell lysates were incubated with Ni-NTA beads, which bind His-tagged ubiquitinated proteins followed by Western blotting using anti-USP17 antibody. Ubiquitination of USP17 was found to be reduced by Mdm2, indicating the ubiquitin-dependent regulation of USP17 levels by Mdm2 (Figure 5E). To check the interaction between Mdm2 and USP17, cell lysates of HEK-293T cells were subjected to coimmunoprecipitation using anti–Mdm2 antibody–bound agarose resin followed by Western blotting. It was found that Mdm2 interacts with USP17 (Figure 5F). To further confirm Mdm2-mediated stabilization of USP17, Mdm2 was down-regulated using specific siRNA in HEK-293T cells. The levels of USP17 were found to be reduced in the presence of Mdm2 siRNA (Figure 5G). These results indicate that Mdm2 increases the levels of USP17 in ubiquitin-dependent manner by interacting with it.

### Human Immunodeficiency Virus Type 1 Vif Mediates Virus-Induced Increase in Expression of Ubiquitin Specific Protease 17

**FIGURE 6:**
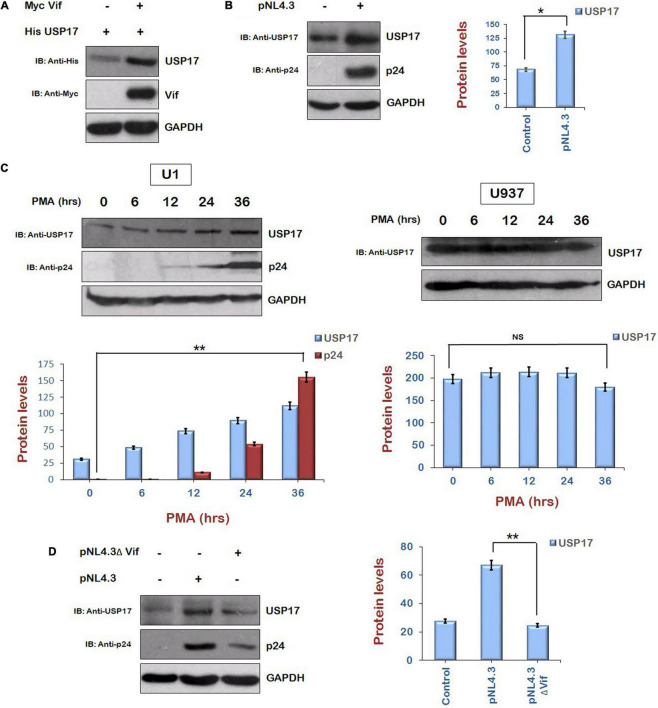
Human immunodeficiency virus type 1 Vif increases the expression of USP17. **(A)** HEK-293T cells were cotransfected with His USP17 and Myc Vif expression plasmids for 24 h. Cell lysates were subjected to Western blot analysis using anti-His, anti-Myc, and anti-GAPDH antibodies. **(B)** HEK-293T cells were transfected with pNL4.3 plasmid for 24 h. Cell lysates were analyzed by Western blotting with anti-USP17, anti-p24, and anti-GAPDH antibodies. **(C)** U1 and U937 cells were treated with PMA (100 μg/mL) for different time intervals as shown. Cell lysates were analyzed by Western blotting using anti-p24, anti-USP17, and anti-GAPDH antibodies. **(D)** HEK-293T cells were transfected with pNL4.3 or pNL4.3ΔVif for 24 h. Cell lysates were analyzed by Western blotting with anti-USP17, anti-p24, and anti-GAPDH antibodies. GAPDH was used as loading control. Densitometry analysis was performed using ImageJ and shown as bar graph with error bars. Data obtained are presented as mean ± s.e.m. of three independent experiments. *p*-value was calculated by two-tailed *t*-test [*p < 0.05, **p < 0.01, NS (not significant)].

As the up-regulation of HIV-1 Tat levels by Vif *via* AKT signaling pathway is mediated by Mdm2-induced enhancement of USP17 levels, the regulation of USP17 levels by HIV-1 and the role of Vif in this phenomenon were also investigated. We transfected His USP17 either alone or with pNL4.3 in HEK-293T cells for 24 h. The expression level of USP17 was found to be enhanced in the presence of virus (Figure 6A). The effect of HIV-1 on USP17 expression was also investigated in U1 cells. U937 cells (parent cell line of U1 cells and contains no virus) were used as control cells for the experiment. U1 and U937 cells were treated with PMA (100 μg/mL) to activate latent virus for different time intervals followed by Western blotting. The expression of USP17 was found to be induced by HIV-1 replication in U1 cells upon PMA treatment, whereas there was no effect of PMA treatment on USP17 protein levels in U937 cells (Figure 6B). These results indicate that USP17 is induced by HIV-1 infection.

To check the role of HIV-1 Vif in inducing the expression of USP17, HEK-293T cells were cotransfected with Myc Vif and His USP17 encoding plasmids for 24 h. Cells were lysed and analyzed by Western blotting. Vif was found to increase the expression of USP17 (Figure 6C). To further confirm the involvement of HIV-1 Vif in virus-induced increase in the expression of USP17, HEK-293T cells were transfected with pNL4.3 or pNL4.3ΔVif for 24 h. Cell lysates were subjected to Western blotting for the analysis of USP17 protein levels. pNL4.3 was observed to increase the expression of USP17 as also shown above, but deletion of Vif abrogated the virus-induced increase in USP17 protein levels (Figure 6D). These results indicate that HIV-1–induced enhancement in USP17 protein levels is mediated by Vif.

### Vif Induces Ubiquitin-Mediated Proteasomal Degradation of Mdm2

**FIGURE 7:**
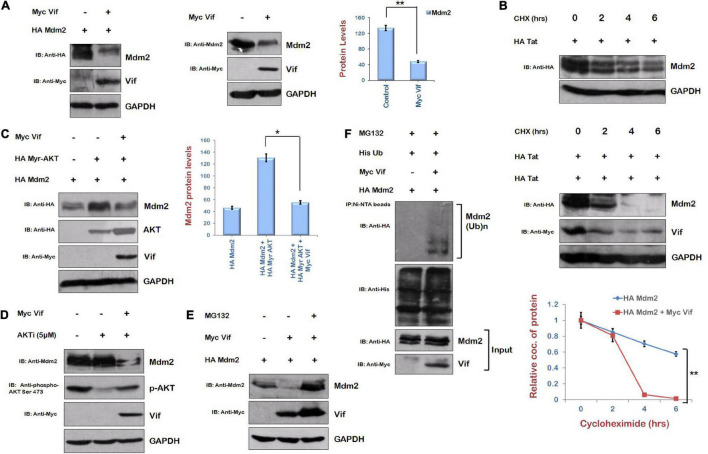
Human immunodeficiency virus type 1 Vif induces proteasomal degradation of Mdm2. **(A)** HEK-293T cells were cotransfected with HA Mdm2 and Myc Vif expression plasmids for 24 h. Cell lysates were subjected to Western blot analysis using anti-HA, anti-Myc, and anti-GAPDH antibodies. HEK-293T cells were transfected with Myc Vif encoding plasmid for 24 h. Cell lysates were analyzed by Western blotting with anti-Mdm2, anti-Myc, and anti-GAPDH antibodies. **(B)** HEK-293T cells were transfected with HA Mdm2 and Myc Vif expression plasmids for 24 h as indicated and treated with CHX (100 μg/mL) for indicated time periods. Cells were lysed, and cell lysates were subjected to SDS-PAGE followed by Western blotting using anti-HA, anti-Myc, and anti-GAPDH antibodies. Densitometry analysis was performed using ImageJ and shown as line graph with error bars. **(C)** HEK-293T cells were cotransfected with HA Mdm2, HA Myr-AKT, and Myc Vif encoding plasmids as shown for 24 h. Cells were lysed, and cell lysates were subjected to SDS-PAGE followed by Western blotting with anti-HA, anti-Myc, and anti-GAPDH antibodies. Densitometry analysis was performed using ImageJ and shown as bar graph with error bars. **(D)** HEK-293T cells were transfected with Myc Vif expression plasmid and treated with AKTi (5 μM) for 24 h. Cell lysates were analyzed by Western blotting with anti-Mdm2, anti-Myc, anti–phospho-AKT Ser473, and anti-GAPDH antibodies. **(E)** HEK-293T cells were cotransfected with HA Mdm2 and Myc Vif encoding plasmids for 24 h. Cells were treated with MG132 (20 μM) for 8 h. Cell lysates were analyzed by Western blotting with anti-HA, anti-Myc, and anti-GAPDH antibodies. GAPDH was used as loading control. **(F)** HEK-293T cells were cotransfected with His Ub, Myc Vif, and HA Mdm2 for 24 h. Cells were treated with MG132 (20 μM) for 8 h. Cell lysates were subjected to immunoprecipitation with Ni-NTA beads followed by Western blotting with anti-HA antibody. Data obtained are presented as mean ± s.e.m. of three independent experiments. *p*-value was calculated by two-tailed *t*-test (*p < 0.05, **p < 0.01).

Mdm2 is the downstream target of AKT. When PI3/AKT pathway is activated, AKT is phosphorylated, and further, it phosphorylates and stabilizes Mdm2 (Feng et al., 2004). As Vif increased the phosphorylation of AKT, and we are showing that Vif increases the levels of Tat protein where AKT signaling pathway plays an important role *via* Mdm2-mediated enhancement of USP17 (which stabilizes Tat by deubiquitination) levels, the effect of Vif on the levels of Mdm2 was also investigated. HEK-293T cells were cotransfected with HA Mdm2 and Myc Vif plasmids for 24 h. Western blot analysis of these cells surprisingly showed that Mdm2 is degraded in the presence of Vif (Figure 7A). Vif was also able to degrade Mdm2 at the endogenous level (Figure 7A). The cycloheximide chase of Mdm2 alone and in the presence of Vif also suggested that the stability of Mdm2 is reduced in the presence of Vif (Figure 7B). These results were confirmed using the constitutively active form of AKT, that is, Myr AKT, which stabilizes Mdm2. HEK-293T cells were cotransfected with HA Mdm2, HA Myr AKT, and Vif for 24 h, and cells were subjected to Western blot analysis. As expected, Myr AKT increased the levels of Mdm2. Vif was found to reduce the levels of Mdm2 even in the presence of Myr AKT, which is itself increased by Vif (Figure 7C). This result was also validated using the inhibitor of AKT activity, that is, AKTi. Vif increased the levels of phospho-AKT Ser 473 in the presence of AKTi as described previously, but the expression level of Mdm2 was found to be reduced in the presence of Vif (Figure 7D).

The mechanism of Vif-mediated degradation of Mdm2 was also investigated. HEK-293T cells were cotransfected with HA Mdm2 and Myc Vif in the presence or absence of MG132 (proteasomal inhibitor). Vif-mediated degradation of Mdm2 was found to be reversed by MG132 (Figure 7E). This indicates the posttranslational regulation of Mdm2 by Vif. The ubiquitination levels of Mdm2 in the presence of Vif were also checked. HEK-293T cells were cotransfected with HA Mdm2, Myc Vif, and His Ub plasmids. Cells were also treated with MG132 to accumulate ubiquitinated species. Cells were lysed, and cell lysate was incubated with Ni-NTA beads, which bind His-tagged ubiquitinated proteins. The ubiquitination of Mdm2 was found to be increased in the presence of Vif (Figure 7F). These results indicate that Vif induces ubiquitin-mediated proteasomal degradation of Mdm2.

## Discussion

There are number of restriction factors that protect the host cells from the pathogen. HIV-1 has evolved various mechanisms to counter these host cell factors. The well-known anti–HIV-1 restriction factors are TRIM5 (tripartite motif 5) (Ganser-Pornillos and Pornillos, 2019), APOBEC3G (Zhang et al., 2003), SAMHD1 (SAM and HD domain containing deoxyribonucleoside triphosphate triphosphohydrolase 1) (St Gelais et al., 2012), tetherin, or BST-2 (bone marrow stromal antigen-2) (Le Tortorec et al., 2011). HIV-1 has developed various strategies to evade these restriction factors except TRIM5 as none of the HIV-1 proteins is known to target this. The restriction factors SAMHD1, tetherin, and APOBEC3G are targeted by Vpx/Vpr, Vpu (or Env), and Vif, respectively (Malim and Bieniasz, 2012). HIV-1 Vif-mediated APOBEC3G degradation (Yu et al., 2003) is essential for viral replication. However, there are very few reports about the regulation of Vif protein. Core-binding factor β (CBF-β) has been shown to stabilize Vif with subsequent effects on APOBEC3G levels (Jäger et al., 2011). MDM2 is reported to promote Vif degradation to elevate APOBEC3G levels. As Mdm2 is a downstream target of AKT (Cooray, 2004; Manning and Cantley, 2007), which is reported to be induced by HIV-1 Tat (Borgatti et al., 1997; Chugh et al., 2001, 2008; Deregibus et al., 2002), we investigated the effect Vif on AKT signaling pathway to find out how HIV-1 copes up with this host antiviral response. Vif was observed to increase the levels of phospho-AKT Ser473. As HIV-1 Tat is already known to activate AKT signaling pathway (Borgatti et al., 1997; Chugh et al., 2001, 2008; Deregibus et al., 2002), and HIV-1 proteins are known to regulate each other by exploiting host proteins (Wang et al., 2008; Lata et al., 2015), the effect of Vif on the expression of Tat and the role of AKT signaling pathway in this process were investigated. Vif was found to increase the levels of Tat protein, and Tat-mediated LTR transactivation was also found to be enhanced in the presence of Vif. HIV-1 Vif and Tat were also found to interact with each other. The inhibition of AKT phosphorylation interfered with Vif-mediated increase in Tat levels. Mdm2, a downstream target of AKT, was found to induce an increase in the levels of Tat protein. When the mechanism of Mdm2-mediated stabilization of Tat was investigated, it was observed that Mdm2 can up-regulate the levels of HIV-1 Tat protein in ubiquitin-dependent manner *via* USP17, which stabilizes Tat by deubiquitinating it. HIV-1 also induced an increase in the levels of USP17, which was observed to be mediated by Vif. Thus, Vif-mediated increase in the levels of Tat protein might be independent of Tat-Vif interaction as it has been found to be mediated by AKT signaling pathway. Surprisingly, Vif induced proteasomal degradation of Mdm2 protein instead of increasing its expression, which was expected from the inducing effect of Vif on phospho-AKT Ser 473. These results indicate the complex role of AKT signaling pathway in regulation of HIV-1 protein expression and viral replication.

**FIGURE 8:**
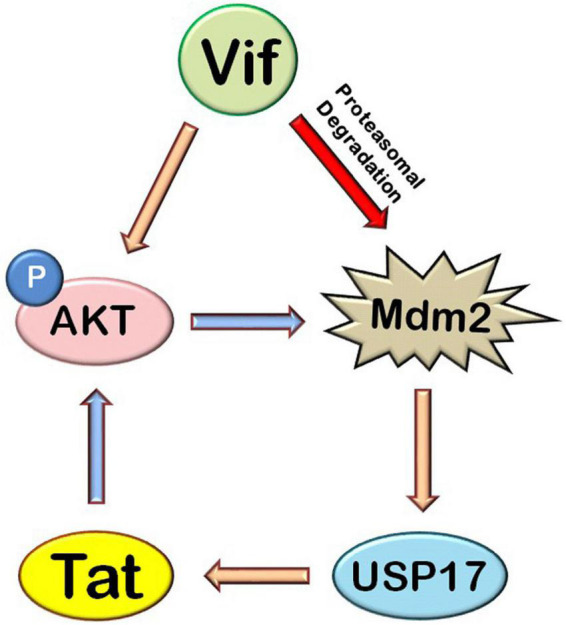
Mechanistic model of HIV-1 Vif–mediated up-regulation of Tat *via* AKT signaling pathway. HIV-1 Tat induces AKT signaling pathway and increases the phosphorylation of AKT and Mdm2. HIV-1 Vif increases the expression of Tat protein *via* AKT signaling pathway where Mdm2 acts as the mediator. Mdm2 increases the expression of Tat by inducing an increase in the levels of USP17, which stabilizes the levels of Tat by deubiquitination. HIV-1 Vif also induces proteasomal degradation of Mdm2 (blue arrows: previously known phenomena, golden yellow arrows: novel inductions, red arrows: novel inhibitions).

The exploitation and modulation of host cellular signaling pathways by HIV-1 proteins are a very complex phenomenon. HIV-1 Tat is an early protein of the virus, which activates AKT signaling pathway (Borgatti et al., 1997; Chugh et al., 2001, 2008; Deregibus et al., 2002). Tat also phosphorylates Mdm2 *via* AKT, resulting in its stabilization as previously shown by us (Raja et al., 2017), and Mdm2 further increases the levels of Tat as shown previously and also increases the LTR transcriptional activity of Tat by inducing its non-proteolytic K63 ubiquitination (Bres et al., 2003), thus creating a positive feedback loop between Tat, AKT, and Mdm2. On the one hand, HIV-1 Vif also increases phosphorylation of AKT, which increases Tat levels and viral replication *via* Mdm2-mediated enhancement of USP17 levels. On the other hand, Vif induces proteasomal degradation of Mdm2 and reduces the levels of Mdm2, which is activated by AKT itself (Cooray, 2004; Manning and Cantley, 2007) and degrades Vif (Yu et al., 2003), creating a negative feedback loop between Vif and Mdm2 (Figure 8). Thus, we hypothesize that HIV-1 exploits AKT signaling pathway through its activation by Tat and Vif so as to maintain the sufficient levels of Mdm2 in the host cell to increase Tat levels and activity, resulting in more viral replication. But when there is more increase in the levels of Mdm2, HIV-1 regulates the expression of Mdm2 *via* inducing its degradation by Vif so as to prevent Mdm2-mediated proteasomal degradation of Vif. It helps the virus to evade the host restriction machinery more effectively and survive in the host cell.

## Data Availability Statement

The original contributions presented in the study are included in the article/supplementary material, further inquiries can be directed to the corresponding authors.

## Author Contributions

SL and VS designed and performed the experiments. All authors conceived the idea, wrote the manuscript, contributed to the article, and approved the submitted version.

## Conflict of Interest

The authors declare that the research was conducted in the absence of any commercial or financial relationships that could be construed as a potential conflict of interest.

## Publisher’s Note

All claims expressed in this article are solely those of the authors and do not necessarily represent those of their affiliated organizations, or those of the publisher, the editors and the reviewers. Any product that may be evaluated in this article, or claim that may be made by its manufacturer, is not guaranteed or endorsed by the publisher.
